# Using patient feedback to adapt intervention materials based on acceptance and commitment therapy for people receiving renal dialysis

**DOI:** 10.1186/s12894-021-00921-5

**Published:** 2021-11-15

**Authors:** James Elander, Romaana Kapadi, Emma Coyne, Maarten W. Taal, Nicholas M. Selby, Carol Stalker, Kathryn Mitchell

**Affiliations:** 1 School of Psychology, University of Derby, Derby, UK; 2 Nottingham University Hospitals NHS Trust, Nottingham, UK; 3 Centre for Kidney Research and Innovation, University of Nottingham, Derby, UK

**Keywords:** Acceptance and commitment therapy (ACT), Tailored intervention, Kidney failure, Haemodialysis

## Abstract

**Background:**

Theory-based intervention materials must be carefully adapted to meet the needs of users with specific physical conditions. Acceptance and Commitment Therapy (ACT) has been adapted successfully for cancer, chronic pain, diabetes, irritable bowel syndrome, multiple sclerosis, and a range of other conditions, but not so far for people receiving renal haemodialysis. This paper presents findings from a study to adapt ACT-based intervention materials specifically for renal dialysis.

**Methods:**

Draft written materials consisting of four stories depicting fictitious individuals who used ACT-related techniques to help overcome different challenges and difficulties related to dialysis were adapted using a systematic patient consultation process. The participants were 18 people aged 19–80 years, with chronic kidney disease and receiving renal dialysis. Individual, semi-structured interviews were conducted to elicit participants’ views about how the content of the draft materials should be adapted to make them more realistic and relevant for people receiving renal dialysis and about how the materials should be presented and delivered to people receiving renal dialysis. The interview transcripts were analysed using a qualitative adaptation of the Delphi method in which themes are used as a framework for translating feedback into proposals for modifications.

**Results:**

The analysis of patient feedback supported the use of patient stories but suggested they should be presented by video and narrated by real dialysis patients. They also indicated specific adaptations to make the stories more credible and realistic. Participant feedback was translated into proposals for change that were considered along with clinical, ethical and theoretical factors. The outcome was a design for a video-based intervention that separated the stories about individuals from the explanations of the specific ACT techniques and provided greater structure, with material organised into smaller chunks. This intervention is adapted specifically for people receiving renal dialysis while retaining the distinctive theoretical principles of ACT.

**Conclusions:**

The study shows the value of consulting patients in the development of intervention materials and illustrates a process for integrating patient feedback with theoretical, clinical and practical considerations in intervention design.

**Supplementary Information:**

The online version contains supplementary material available at 10.1186/s12894-021-00921-5.

## Introduction

Over 28,000 people with end-stage renal disease receive dialysis in the UK and over 25,000 receive hospital-based haemodialysis [1], which involves direct blood filtration by a dialysis machine for 4 h per treatment — delivered 3 times per week. Haemodialysis can extend patients’ lives, but it causes significant cardiovascular morbidity and reduced quality of life [2], and is also expensive, costing over £27,000 per patient per year and consuming approximately 1.3% of all NHS spending [3]. Commencing and living with haemodialysis involves multiple losses and stressors [4, 5], and people receiving haemodialysis face significant challenges to adjust to the demands of treatment and cope with side effects and complications, which makes many dialysis patients vulnerable to impaired wellbeing and quality of life [6, 7].

It is therefore important to maximise the benefits obtained by dialysis and improve patients’ experiences of treatment, as well as to help people receiving dialysis achieve and maintain the best possible quality of life. Psychological interventions can contribute to this but need to be specifically adapted to address the unique challenges of kidney failure and treatment by dialysis. For example, one study used a review of evidence about factors associated with psychological distress among people with end-stage kidney disease to guide the formulation of a cognitive behaviour therapy protocol that was specifically adapted for people undergoing dialysis [8].

Acceptance and Commitment Therapy (ACT) is a mindfulness-based behavioural therapy whose goal is to enable people to lead rich and meaningful lives while accepting the pain and suffering that inevitably go with that [9]. ACT uses metaphor, paradox, mindfulness skills and experiential exercises to promote psychological flexibility, which involves being open, aware and in contact with the present moment, and engaging in activities consistent with one’s goals and values [10–12].

A review of meta-analyses including 133 randomized-controlled trials concluded that ACT is efficacious for all conditions examined [13] and ACT interventions have improved self-management, treatment adherence and adjustment among people with chronic physical conditions including cancer, cardiac disease, type 2 diabetes, epilepsy, multiple sclerosis, cerebral palsy, and paediatric brain injury [14].

ACT has considerable potential for application to people receiving renal dialysis [15], and a preliminary case study of ACT delivered individually to a person with end-stage renal disease suggested the approach had potential for improving adherence to dialysis [16]. However, ACT interventions must be carefully adapted for each condition, based on evidence about what aspects of content and delivery are most relevant for each patient group. For example, it is important to contextualise ACT-related issues and processes using realistic condition-specific examples. Factors specific to dialysis that need to be considered include the burdensome nature of dialysis, the specific ways it limits the lives of affected people, and the cognitive impact of dialysis itself on attention, thinking and memory. The present study therefore aimed to consult people with experience of receiving haemodialysis about the specific ways a prototype ACT intervention should be adapted to make it as relevant and useful as possible for people receiving haemodialysis.

Adaptations of ACT interventions need to consider both content and delivery. An adaptation of ACT content for diabetes involved incorporating behaviours relevant to diabetes adherence (mindful eating, exercising, glucose monitoring and administering insulin injections) into a generic creative hopelessness ACT exercise [17]. Other adaptations involved generating new condition-specific content; one study generated fictional people with irritable bowel syndrome who were composites of statements posted in an online forum [18].

A very common adaptation of delivery for people with chronic pain is to make the ACT intervention ‘self-help,’ usually by providing materials and instructions for exercises online or in book form, with little or no therapist involvement [19–21]. One study piloted a telephone-supported self-help ACT intervention for haemodialysis patients with depression, using an existing self-help manual [22]. The participants (haemodialysis patients with high depression scores) were instructed which chapters to read each week and had a weekly telephone call from the researcher to provide support in understanding the materials and encourage adherence. However, low recruitment numbers and poor adherence to the self-help manual showed that the intervention was not viable in that form; only one participant out of four completed all the chapters and health problems were the main obstacle to completion [22, 23].

Some adaptations of delivery have focused on the cognitive demands of interventions. In one study, the delivery of an ACT intervention was adapted for women with multiple sclerosis to take account of participants’ cognitive difficulties. The adaptations included using a structured approach to reduce the load on working memory, and managing information with chunking and repetition, diagrams, written summaries and a cartoon drawing [24]. In an adaptation of ACT for people with psychosis, experiential exercises were kept brief and learning points were carefully paced and scaffolded to accommodate cognitive difficulties. The ‘passengers on the bus’ metaphor was presented first as a story and later acted out by facilitators and participants, and a scripted video was used in which a character played by an actor described the challenges in his life, to illustrate the real-world relevance of the metaphor [25].

It is vitally important to involve patients or potential end-users in the adaptation process, which can contribute to the achievement of greater personalised care, which means that “people have choice and control over the way their care is planned and delivered, based on ‘what matters’ to them and their individual strengths, needs and preferences” [26, p. 6, see also 27].

In one study, ACT was adapted for cancer-specific concerns by obtaining feedback from participants as well as ACT experts during the intervention development [28]. In another, a paper-based prototype of ACT for prevention of weight gain was tested in focus groups and interviews with members of the target audience, followed by expert review to ensure fidelity with the underlying theory [29]. In another, text for an ACT intervention for the partners of people with cancer was evaluated by potential users before it was further developed [30]. The present study followed good practice in adapting ACT-based materials for dialysis by conducting a systematic patient consultation exercise to obtain feedback about the content and delivery of prototype materials in order to inform the next stage of development of the intervention.

## Methods

The study involved semi-structured interviews with a sample of people who were selected to take part because of their expert patient experience of treatment for chronic kidney disease with renal haemodialysis.

### ACT-based draft materials

The draft materials consisted of a printed booklet of four case descriptions of fictious dialysis patients that illustrated different difficulties and challenges associated with renal disease and in-centre dialysis treatment, and ways in which the fictitious patients were helped to overcome those challenges by using techniques based on ACT (see Additional file 1). The draft materials were closely based on ACT principles and practice and were prepared in collaboration with a consultant clinical psychologist who had extensive experience of delivering ACT therapy (EC), and with clinicians who had extensive experience of haemodialysis treatment for chronic kidney disease (MWT and NMS).

### Participants and recruitment

The participants were 18 people with end-stage renal disease who were aged over 18 years, had received in-centre dialysis for at least 90 days in the last two years (including patients who were presently on home haemodialysis but had previously received in-centre haemodialysis), and were able to converse in English. People with active infection or malignancy or whose medical condition made participation in an interview difficult were excluded. Key features of study participants are given in Table 1. Participants are identified by pseudonyms.

**Table 1:** Participant information

Participant^a^	Age	Gender	Ethnicity	Marital status	Employment status	Region	Interview method	Current type of haemodialysis	Approx. time on dialysis
1. Adam	52	Male	White	Single	Unemployed	North West England	MS Teams	In-centre	6 years
2. Albert	73	Male	White	Divorced	Retired	East Midlands	MS Teams	In-centre	25 years
3. Simon	43	Male	White	Single	Employed full-time	East of England	MS Teams	In-centre	19 years
4. Arjun	42	Male	Asian/Asian British	Married	Employed part-time	South East England	MS Teams	Home	5 years
5. George	61	Male	White	Married	Retired	North East England	Telephone	In-centre	2 years
6. Oliver	35	Male	White	Married	Unemployed	East Midlands	Telephone	Home	2 years
7. Sarah	19	Female	White	Single	Unemployed	East Midlands	MS Teams	In-centre	7 months
8. Richard	60	Male	White	Single	Retired	East Midlands	Telephone	In-centre	6 years
9. Henry	71	Male	White	Single	Retired	East Midlands	Telephone	In-centre	5 years
10. Helen	51	Female	White	Married	Retired	East Midlands	Telephone	In-centre	6 years
11. Hardeep	80	Male	Asian/Asian British	Widowed	Retired	East Midlands	Telephone	In-centre	7 years
12. Alison	57	Female	White	Single	Unemployed	East Midlands	Telephone	In-centre	6 years
13. Steven	52	Male	White	Married	Retired	East Midlands	Telephone	In-centre	6 years
14. William	73	Male	White	Divorced	Retired	East Midlands	Telephone	In-centre	6 months
15. Diane	68	Female	White	Widowed	Retired	East Midlands	Telephone	In-centre	1 year
16. Wilfred	87	Male	White	Widowed	Retired	East Midlands	Telephone	In-centre	1 year
17. Arthur	78	Male	White	Widowed	Retired	East Midlands	Telephone	In-centre	6 years
18. Brian	79	Male	White	Widowed	Employed part-time	East Midlands	Telephone	In-centre	14 years

^a^Pseudonyms are used to identify participants

Participants were recruited from patients attending the Dialysis Unit, Royal Derby Hospital, and by posting brief invitations to participate on the Kidney Care UK and British Renal Society online support groups and Facebook groups, and on other Facebook groups including Transplant and Dialysis Support Group, Kidney Research UK, Dialysis Network UK, Kidney Care UK’s Young Adult Kidney Group and Chronic Kidney Disease UK. There were twenty-eight individuals who responded initially to the invitation, who all met the inclusion criteria and were eligible to participate, of whom ten were not able to arrange an interview, so there were eighteen who took part in an interview. Recruitment and data collection took place between April and September 2020.

### Procedure

All the study methods were carried out in accordance with UK National Health Service and British Psychological Society guidelines and regulations. Written informed consent was obtained from all participants. Participants recruited at the hospital were given paper copies of the participant information sheet and the booklet of draft materials at least 48 h before being asked for written informed consent, after which a convenient time was arranged to conduct a telephone or videocall interview. Participants recruited online were directed to a brief online survey hosted by Qualtrics, a secure online survey platform, where they viewed further information about the study and provided brief information about eligibility, informed consent, and availability for interview by telephone or online videocall using Microsoft Teams. The draft materials were then emailed to participants and a convenient time was arranged to conduct the interview, allowing at least 48 h between recruitment and interview. All the interviews were conducted by RK.

The interviews were up to one hour in length and followed a prepared semi-structured interview schedule. Participants were asked how the case descriptions could be adapted and improved to make them more meaningful, relevant and useful for in-centre dialysis patients, and how materials like these should be presented to dialysis patients for the most convenient and effective use. The interviews were audio-recorded and transcribed verbatim. The transcripts were anonymised so they contained no personally identifying information and participants were given pseudonyms.

### Data analysis

The interview transcripts were analysed using an adaptation of the Delphi method that employs qualitative data to elicit and summarise expert opinion [31]. The first step was to sort transcript material into pre-identified categories related to specific aspects of the draft materials, which included the overall credibility of the stories, the number and length of the stories, the structure of the stories, specific features of each individual story, suggestions for new content in the stories, suggestions about techniques and strategies portrayed in the stories, and suggestions about how the stories should be presented and delivered. Within each of these categories, participants’ views were organised into themes using a descriptive thematic analysis [32]. The resulting themes were then used as a framework for translating participant feedback into proposed modifications to the materials. An ACT-trained therapist (EC) oversaw the translation of participant feedback into intervention design to ensure formally correct ACT-based psycho-educational materials with content, format and delivery methods modified to optimise benefits for patients receiving dialysis.

## Results

### Analysis of participant feedback

In the narrative analysis below, we present each theme together with representative statements that are extracted verbatim from the interview transcripts. A larger dataset of representative participant feedback is available in Additional file 2. In the second stage of the analysis, described afterwards, we present the ways each of those themes was translated into specific proposals for modifying the materials.

#### Theme 1: the stories were credible

The first theme reflected the majority view that the stories were generally credible and relatable. Eleven participants specifically commented on this aspect of the stories, for example:

“Yes, they were very good actually and some of it is spot on” (Arjun).

“I found a little bit of my situation in each of them, and when I did that I thought, oh, so, how would I react and how would I behave and would that benefit me, you know” (George).

“It's pretty much how things are I think” (Richard).

“I know they’re fictional, but they probably could be real cases because people do suffer and like I say especially at the moment with things as they are um I think the mental health problems are far greater than perhaps they would've been” (Diane).

“I could relate to all of them” (Brian).

Three participants reported finding the stories artificial or unrelatable and these three provided 12 statements about this issue. The perception seemed to arise from the fact the stories were presented as fictional, and two of the participants suggested using true stories from real patients:

“I don't know why they have to be made up. I don't know why they can't just be real stories from real people” (Oliver).

“Yeah, but couldn't you get say a person like myself who wouldn’t mind, get their story which is actual facts” (Steven).

#### Theme 2: don’t sugar-coat dialysis

Four participants made eight statements describing the view that the stories should not be unrealistically positive and should reflect some of their own very difficult experiences with dialysis:

“Unfortunately, sugarcoating stories I don't think is gonna help some people because you know, some people need to be told you must do this otherwise you're gonna die …” (Oliver).

“I found the first month or so absolutely horrific. I really did and I couldn't see anything in the stories that I could relate to, err regarding my initial start” (Arthur).

#### Theme 3: don’t always make the first thing they try work out

Participants generally appreciated the narrative structure of the stories in which the characters encountered difficulties and then found ways to overcome them, but there were four participants and nine statements that offered the view that it seemed unrealistic that in each case the first strategy suggested worked:

“… the way that they work they sort of go down and then to a thing and then start climbing up the other side which is the way dialysis should be …” (Albert).

“… it would be worth doing one whereby the first solution that maybe the renal psychologist or maybe the consultants suggested or maybe the nurse suggested didn’t work but because they’d made that effort they were then able to go and talk to someone to find another way to try and sort it out” (Adam).

#### Theme 4: psychologists are not so available

There were five statements from three participants who pointed out that patients on dialysis would not have such easy access to a psychologist:

“… in my dialysis unit we have a renal counsellor but we don’t really have a psychologist” (Simon).

“I was like really, really low, um and the guy in there said to me, like oh I'll refer you to a psychologist, but I never got referred to one” (Sarah).

“I've never seen a psychiatrist or psychologist” (Brian).

#### Theme 5: involve other people

Two of the participants commented in four statements that the stories should show people getting help and support from others:

“… what is it like to live with a dialysis patient because it’s not a journey just for one person, it could be a journey for the partner, or family member that helps, or friend that helps” (Arjun).

“I think help from other people is very, very important, whether you've got a, uh, a partner or family at home, or you can see a professional” (George).

#### Theme 6: realism in individual stories

Participants made specific suggestions for amending details to make the stories more realistic. These included representing minority ethnic groups and depicting more positive aspects of the lives of people receiving dialysis:

**“**I’m a Hindu and you know when I had kidney failure for my parents it was hard to take and being an Indian is very different to being a Caucasian in all aspects of life and I think that’s true whether you’re a Hindu, Muslim, a Sikh or whatever. I think on general broad terms in an Asian community we have a very different way of life …” (Arjun).

“… show that you can have local holidays in your country, you can go shopping, you can go for dinner, these things are all positive and we need to show that …” (Arjun).

“… you have to look at other things to look forward to. Like going out for a meal or something you know or going out for a day when you're not on dialysis” (Steven) .

Several participants suggested that Keiron’s work situation was not very credible, although others thought it might occur sometimes and that Keiron’s story had a valuable message:

“I think workplaces would understand that, and I don't think they would be so pushy to um, you know, call him while, call someone while they're on a session and get them to work, and especially like quite frequently” (Sarah).

“… it was probably through ignorance that his bosses didn't understand what was happening to him, didn’t realise that it was vital that he had treatment or else he’d die” (Diane).

“…I felt it was quite sad but I've been there, done that …” (Alison).

Several participants identified with Joanne’s story, and there were no suggestions of specific changes to make about that story:

“[The story] where they’re struggling with the fact that they have to do this and it’s not very pleasant, it’s the woman who’s got the two kids […] that story rings true […] there’s a big problem with people not understanding what the treatment is, so even though you include people in the treatment they just don’t get it” (Adam).

“… those characters I identified with a couple of them, you know, you can you feel a bit sorry for yourself or feel down and so you seek comfort in junk food or something” (George).

Participants also supported the story of Margaret, and suggested expanding the ways in which Margaret was portrayed doing things for herself, as well as suggesting Margaret’s family should not be so free to visit her during dialysis:

“I think one of the best outcomes you can have on dialysis is if you do everything yourself … even if you’re not going to needle yourself … just coming in and lining your machine up and getting everything ready is an element of control that makes life a lot easier, so that might be worth adding into that story with her” (Adam).

“… unless someone's a family member [and] is coming in to actually help to dialyse you … I found that a very strange thing in the story that family started to pop in (George).

Many participants identified with David because they were a similar age, but suggested it seemed incongruous that David was previously a car mechanic but did not have a car:

“Yeah, well I think that and because in the early days I was very similar, like I was very impatient and very intolerant at the start. Err, I'm not now I've mellowed considerably” (Arthur).

“… he was a car mechanic, retired, but he didn't seem to have a car in itself, so we went with transport. […] in his case I just thought it was a bit strange that he was, it was, uh, it was an inappropriate choice of profession. He’s a car mechanic and likes to fix peoples cars, but he doesn't drive himself” (George).

#### Theme 7: focus more on the techniques

There were six participants who provided eight statements in which they identified and supported the ACT techniques that were portrayed, but also suggested the techniques should be presented carefully:

“I liked the one about the trains and getting on the old um, you know, I’m cross with everyone train” (Simon).

“I think that was in one of their stories, you know, like gathering your thoughts and like tackling them one at a time … Um, I think another one was set like setting goals for themselves” (Sarah).

“Writing down my thoughts, I don’t know, it did help me” (Hardeep).

“I appreciated that because I keep a diary, that's one of the things I learnt at previous dialysis was to keep a diary” (Alison).

“… a lot of the people you’re dealing with in hospital, I think they would have to be shown these techniques very, very sort of gently and carefully” (Brian).

#### Theme 8: use video format

Nine participants provided 12 statements commenting on the length of the stories and the time and effort needed to read them in the form of text, and suggested that video could be a better way of presenting the stories, including suggestions about using cartoons:

“as text it all, it was just too long. But if you are planning on doing it as a video then it wouldn’t really matter …” (Oliver).

“I do think actually as well that sort of visual video or audio or whatever is a lot better than written” (Richard).

“I mean you could try something humorous with animation …” (Adam).

Other perceived advantages of video format were that the stories could be viewed during dialysis sessions and that people could watch together:

“Provide a tablet and this clamp and you can just, any patient can then watch it, clamp it to the table …” (Albert).

“I'd watch it during dialysis definitely” (Sarah).

“… then if they could watch something at home as well, because then if they've got, if they’re living with family and stuff, wives, kids or whatever, or parents or whatever, then they can share ideas can’t they” (Richard).

### Translating feedback into proposed modifications

Each of the themes was then translated into proposed changes to the intervention materials. First, we assessed the suggestions made by participants within each theme, taking account of the degree of consensus among participants and how representative participants were of the wider potential audience. Second, we identified a set of potential changes and evaluated these taking into consideration ethical constraints, practical considerations, and the need to maintain consistency with ACT theory. We also had to consider any potential impacts of modifications on the pedagogic integrity of the intervention materials, so modifications that would have reduced the integrity of the materials as psycho-educational tools were avoided. Table 2 gives an overview of the proposals for modifying the materials associated with each theme.

**Table 2:** Proposed modifications for the draft materials

Themes	Proposed modifications
1. The stories were credible	Retain the concept of using individual stories
	Present the stories as real rather than fictitious, but still script them so that we can control the content and so that details of real-life patients are not revealed
	Have real patients narrate the stories
2. Don’t sugar-coat dialysis	Include a bit more recognition of the gritty realism of dialysis (including amending the presentation of Margaret’s efforts to present dialysis only in a positive way to her children)
3. Don’t always make the first thing they try work out	Have some description of things that were not successful at first—possibly related to some of the gritty realism about dialysis and the non-availability of psychological support (see themes 2 and 4)
4. Psychologists are not so available	Make it more credible how people are offered help, and give renal counsellors or other staff, rather than psychologists, a bigger role in the stories
5. Involve other people	Make some of the characters’ family and friends more visible in the analysis
6. Realism in individual stories	Have at least one character from a minority ethnic group
	Amend or explain some details of Keiron’s work situation
	Amend the details of Margaret’s family visiting her on dialysis (but keep her family visibly involved—see above)
	Give David a different occupation so his dependence on hospital transport is more credible or explain why he does not drive to dialysis
7. Focus more on the techniques	Separate out the techniques from the individual stories—giving more room within the stories for realism etc. and enabling ‘technique’ videos to be viewed separately and repeatedly
8. Use video format	Use video but with a cartoon format to preserve participant anonymity
	Use the Toonly© system to present scripted narratives, spoken by real patients

The translation of participant feedback into changes to the materials often involved simultaneous consideration of multiple factors. For example, although participants overwhelmingly supported the concept of using stories about individual people’s experiences, several participants suggested using real stories told by real patients, possibly prompted by the fact the stories were presented as ‘fictional’. We considered obtaining true stories told by real-life people receiving dialysis, but also had to consider potential ethical issues, for example to protect individuals who might potentially be identifiable, and the need to maintain consistency with ACT theory. We therefore decided to retain control over the content of the stories but present them as true stories and include examples and issues based on real patients where possible (in fact, many elements of the original stories were based loosely on real-life patients and incidents). We also decided to recruit real patients to narrate the scripted stories and to match narrators to stories as far as possible so that narrators presented stories that were close to their own personal experiences.

We also had to balance participants’ feedback about presenting more of the gritty reality of dialysis against the need for the materials not to cause distress or anxiety, especially considering that potential users could include people who were new to dialysis or had not yet started dialysis. However, we could make small changes to alter the balance between realism and positivity, several of which were specifically suggested by participants. For example, one story described how Margaret’s family “started to pop in to see her on dialysis,” and a participant suggested how the wording could be changed: “*I'd certainly tweak that sentence. You know, family popped in. I would change it to something like, you know, family started to ask more questions*.” (George).

There were several other points where participant feedback suggested that changes to wording and presentation would improve the realism of the stories, including the points about not everything a person tries working out first time and about the availability of psychological support. However, around two-thirds of participants were recruited from a clinical centre where there is not a clinical psychologist routinely available, and this may have influenced some of the feedback. We therefore reviewed the depiction of psychological support and gave more prominence to professionals who were not psychologists, but we did not remove the references to psychologists in two of the four stories. We also had to consider whether specific points raised by participants (for example, Keiron’s work and David’s driving) should be addressed by changing details of the stories or by giving more explanation of the circumstances. In some cases, we decided to explain or clarify elements of the stories rather than make the specific changes suggested.

When considering changes to the stories we also had to consider the implications of the change from written text to video, taking account of the need to preserve the integrity of the materials as educational tools. This meant that the stories needed to be shortened and simplified, so it made sense to separate the techniques from the stories, which meant that although the techniques would be mentioned in the stories, a fuller explanation of the techniques and how to use them was provided separately.

We decided to use Toonly© for both the story videos and the videos explaining the techniques. Toonly© is software to produce video recordings with cartoon animations and audio soundtrack. For the stories, this enabled us to use scripted narratives recorded by real dialysis patients but without the narrators appearing visually—they would be ‘spoken’ by a cartoon figure. This preserved the anonymity of the people who narrated the stories and meant that their task was simplified by not having to present themselves visually. We aimed to make the stories as realistic as possible by adjusting the content in response to participants’ feedback and by choosing narrators who identified with the character they were narrating and could tell that story in an authentic way. Narrators were given the stories to read before agreeing to act as narrators so they could decide whether they felt ‘right’ telling that story. Using cartoon animation to present the stories was also consistent with ACT theory. Using cartoon characters to present ACT-based content helps with the use of metaphor and cognitive diffusion, enabling viewers to detach more easily from the literal use of language [9, 33].

One aspect of the feedback that was important for us to implement was making the characters portrayed in the stories include minority ethnic groups. For that reason, we changed two of the characters’ names, with ‘Keiron’ becoming ‘Jayden’ and ‘Joanne’ becoming ‘Naadiya’, so their names would be consistent with visual identities portraying people from minority ethnic family backgrounds.

**Fig. 1:**
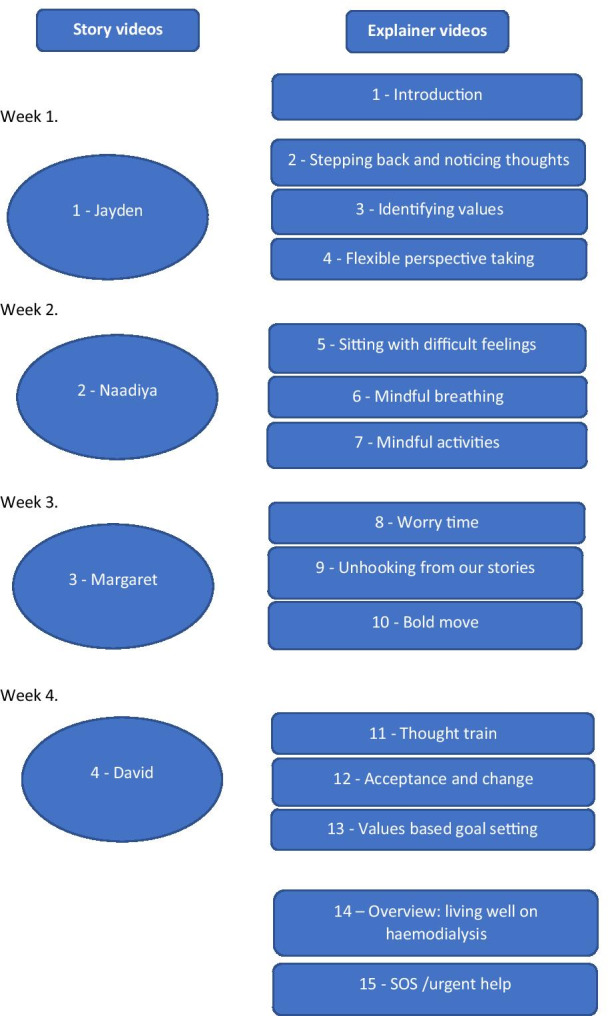
Structure of the adapted ACT intervention

For the techniques, using Toonly© enabled us to produce short presentations of ACT techniques with audio explanations by a clinical psychologist and ACT therapist, and the explainer videos were designed for repeated viewing. Separating the explanations of the techniques from the stories also enabled us to structure the materials into a programme of videos to be watched over four weeks, with one story video and three accompanying explainer videos to be watched each week, plus an introduction to the whole programme and an overview about living well on dialysis and advice about what to do in cases where urgent help was needed (see Fig. 1). The whole package of video materials could be delivered online and viewed on a range of different devices, including in dialysis units and in people’s homes.

## Discussion

The study showed how patient consultation can help to make intervention materials more credible and relatable, and how patients can make important contributions to the development of interventions in a way that is consistent with the principles of personalised care [26, 27]. Participants supported the concept of telling stories about individuals and their experiences and they recognised many of the issues and situations presented in the stories. Participants did not agree on every point and made some contradictory points about the stories, so the adaptation inevitably could not incorporate all the feedback obtained. Also, patients’ views are important, but they are not the only consideration; participants’ feedback had to be moderated by the need for the intervention to be consistent with ACT theory and clinically and ethically acceptable as well as psycho-educationally effective.

The consultation exercise led to a design for an ACT intervention that has many elements in common with previous adaptations of ACT for adult populations, for example the use of composite fictional characters [18], the use of scripted video depicting characters played by actors [25], and the use of stories and cartoons to help explain metaphors and encourage multiple perspective-taking [24, 34]. The resulting intervention should be more acceptable to people receiving dialysis than interventions using written material, which were shown not to be viable in that form [22, 23].

The study did have some limitations, however. It involved a purposive sample of people who were all relatively experienced with haemodialysis. Many of the participants were recruited from the same clinical centre, and minority groups and non-fluent English speakers were under-represented. When the intervention is further developed it will be useful to evaluate it with more diverse samples, including people with less experience of haemodialysis. Future evaluations might include comparing this self-paced mode of delivery with more clinically oriented applications of ACT [16] or assessing the suitability of the intervention for different groups of dialysis patients, some of whom may be depressed. A previous self-help ACT intervention that was specifically designed for haemodialysis patients with depression was not successful because of poor recruitment and adherence [22, 23], but the present intervention is not specifically designed for people with depression and aims to help dialysis patients more generally to improve their adjustment and live better with dialysis. The next step in the present programme of research is to produce the intervention presented in Fig. 1 and conduct a feasibility trial in preparation for a larger randomised trial.

## Conclusions

The study showed how patient feedback can be systematically collected and analysed to inform the adaptation of ACT intervention materials for people affected by specific conditions or treatments. This is especially important for people receiving renal dialysis, which makes unique demands of patients and requires specifically adapted intervention materials, and is also especially important for ACT interventions, which are theoretically distinct and require a carefully controlled and balanced process to incorporate user feedback while ensuring theoretical consistency.

## Supplementary Information

**Additional file 1. **Booklet of draft materials.

**Additional file 2. **Dataset of participant feedback.

## Data Availability

The data analysed during this study are included in this published article and the supplementary information (Additional file 2). To request the data, please contact the first author (JE).

## Abbreviation

ACT: Acceptance and commitment therapy
