# Supplementary material for: Using patient feedback to adapt intervention materials based on acceptance and commitment therapy for people receiving renal dialysis
Source: BMC Urol. 2021 Nov 15;21:157. doi: 10.1186/s12894-021-00921-5 (PMC8591942; doi:10.1186/s12894-021-00921-5)
Supplement: Supplementary file 1 — Additional file 1. Booklet of draft materials. [file 12894_2021_921_MOESM1_ESM.pdf]

Using ACT to help people live well on dialysis:
fictitious patient stories

The following four descriptions are of fictitious (not real) people who found haemodialysis very difficult and struggled to get used to it. They are made up of lots of different people's experiences. The people in the stories were supported to use certain strategies to adapt better to their treatment and live better with dialysis. **We are seeking your opinions and advice about how these descriptions could be improved to make them more realistic, relevant, clear, useful or helpful. You are one of a number of people having dialysis whom we have approached to read the patient stories and then be interviewed about them by a researcher.**

Please remember that these are not typical cases of people on dialysis, and only refer to situations in which people are having haemodialysis in hospital (in-centre haemodialysis). Most people adapt well to dialysis and don't have any major difficulties. However, some people receiving dialysis do have problems, and we are trying to produce information and materials that can be used to help health professionals talk with people about the challenges of dialysis and how people can adapt to those challenges in order to live as well as possible on dialysis.

The patient stories described below have therefore been prepared to highlight some of the difficulties that people on dialysis may have, and show examples of things which have been found to help people overcome these problems. The approaches to overcoming difficulties like these, and the techniques the patient stories highlight, are drawn from an approach known as Acceptance and Commitment Therapy (ACT), which we would like to adapt for people receiving dialysis.

In the next part of the study, we would like to present the stories as short films where people would tell each part of their story. We want to see whether watching the stories is helpful. Before we film the stories, we wanted you to read the stories and give us feedback. In the interview you will be asked about how realistic you think the stories are and if they could be changed to make them a more realistic portrayal of people's lives on dialysis. During the interview we will also ask you about what you think about using short films and whether this would be better than reading the stories. We are also interested to hear what you think about how these and other materials should be presented to people on dialysis.

If you have any questions about the draft stories before the interview, please contact the study team.

Story 1 – 'Kieron'

Kieron is 25 and lives with his mum and dad. He has a job in sales, where the pay is earned by commission on a zero hours contract, so he can't afford to take time off. He hasn't had any long-term health issues previously. His aims in life are to earn lots of money, to be able to move out into his own place and get a car. He likes his job, having his own money and doing his own thing, so he was very shocked when he found out he had severe kidney disease and had to start dialysis straight away.

Part 1. Struggling and getting stuck

Being on dialysis was a massive change for Kieron, as he just wants to get on with his life. Often it is busy at work and he needs to be there, so sometimes he just doesn't turn up for dialysis.

Sometimes, he will get a phone call from work asking him to go in whilst he is having dialysis, and then he just leaves the unit early and does not have the full dialysis treatment. Staff are very worried that he is missing a lot of dialysis but he ignores them as he has 'heard it all before'. They try to talk to him about the risks he is taking but he says 'nothing bad has happened'. The unit staff are very concerned that he is underestimating the risks of missing dialysis. His blood results suggest that he isn't taking his medication regularly and he openly admits he often forgets to reorder his medication and just stops taking it.

Kieron is often very fluid overloaded as he isn't doing enough dialysis and feels unwell, and this makes it harder to keep on top of his job. In the short-term, he thinks putting work first is helping him to progress his career but in the long term it isn't helping his health.

His doctor says to Kieron that he needs to talk to someone about his situation as they are worried about him.

Part 2. Managing difficult thoughts and feelings

Kieron agreed to speak to a Psychologist who suggested to him that he finds it really difficult to say 'no' to work and prioritise his own health. Kieron admitted he wanted to say 'no' to work but when he gets a call, it gives him a panicky feeling and he responds without thinking, usually promising to follow something up or get on with something that needs doing. His mind tells him 'you must keep working or you will lose your job' and 'you just can't say no to work'.

Kieron was asked to try and just notice what his mind was saying to him rather than just react: 'my mind is saying I must say yes to work when they ring me'. He was surprised that just noticing what his mind said let him take a step back from it.

The Psychologist wondered what it would be like if Kieron stopped and checked out what his mind was saying before he acted on it and what it would be like if he said 'no'. The thought that he could say 'no' to work caused Kieron feelings of anxiety. Kieron thought he would feel so terrible he might pass out or have a panic attack if he actually said 'no'. The following week he had just started a dialysis treatment and the phone rang asking him to go in to work ASAP. He didn't immediately respond, but stopped and checked out what his

mind was saying. His first thought was ‘I must go into work’. He then was surprised to notice his mind was saying ‘you could go in but what if you said you would go in after dialysis?’ He took a deep breath and told work that he was on dialysis but could get there that afternoon. He was very surprised to find that instead of feeling overwhelmed he felt okay. The thought of what it might feel like was worse than what it actually felt like.

He started to use the stepping back technique and notice what his mind was telling him. He realised that his mind wasn’t always ‘right’ but often just wanted him to take an easy route.

Part 3. Who am I now? Living in the present

Kieron had always seen himself as a hard worker and didn’t want anyone to see him as ‘ill’ or not progressing in life. He compared himself to his friends who he felt were more successful. He felt if he talked about being ‘ill’ at work people would see him in a negative way. In a session with the Psychologist he was asked to describe a scene from his favourite TV show. He was then asked to tell the story again from the point of view of different characters. He noticed that people could see the same situation very differently depending on their point of view. He was then asked to imagine how his family, friends, people at work and the dialysis unit staff saw him and his situation. He found this exercise quite surprising when he realised that people could see his own situation in different ways too. While he saw himself as a hard worker, other people saw him as a risk taker. He was so focused on working hard that he hadn’t seen himself as a risk taker.

The Psychologist wondered if Kieron was spending a lot of time thinking about the future. Kieron said he was constantly comparing his situation to what he had imagined his future would be and to what his friends were doing who had gone to university or gone on to have successful careers with good salaries. The Psychologist encouraged him to focus on the ‘now’ rather than on his imagined future or comparing himself to others. He started to write down what had gone well at the end of each day. He was really surprised that he was missing out on enjoying some of the good things that were happening in his life because he was so focused on how bad his situation was.

Part 4. Living with purpose: taking action

Kieron was asked by the Psychologist to think about his values and the sort of person he wanted to be. He was asked to look at a list of values which are important to people. He always thought work, money and a car were important to him. He was surprised when looking through the list of values to find that he valued his health and education. He talked about having lots of regrets that he hadn’t stayed on a college course and had gone straight into work.

Instead of thinking about focusing on his job, Kieron decided to focus on two of his values of ‘staying healthy’ and ‘developing himself’. He set some goals in line with these. These were really bold moves for him and scared him, but he decided he needed to stop doing the same thing over and over again and think about the longer term.

He spoke to his GP about his situation. He realised he felt so unwell and that his work was ‘almost killing him’ because he was missing so much dialysis. His GP felt he wasn’t well

enough to work in his current job at the moment. He saw a benefits advisor about his options to support himself. He stopped work and focused on doing his dialysis and taking his medication and getting in better routines. Instead of seeing this as a step backwards, he contacted colleges about courses he could do to progress himself academically.

Kieron decided to use his time on dialysis to study, and his attendance improved. He started to use an app on his phone to help him remember to take his medication. When he had moments where he compared himself to his friends, he stopped and reminded himself that he is working in line with his values of health and education. He is now looking forward to finding a job after his course finishes but is determined that dialysis will be his priority. He also hopes he may have a transplant in the future.

He says although he is on dialysis, it doesn't need to stop him moving forward.

Story 2 – 'Joanne'

Joanne is 40 and lives with her husband and their two daughters. Joanne works part-time as a shop assistant on the checkout in a supermarket. When she was younger, she had a lot of urine infections and missed a lot of school so she didn't do very well at school as she was often in hospital. She has always prioritised family and having children so was delighted to have a family.

Joanne's kidney disease began slowly and got worse over time, so she had time to plan her dialysis as her kidney function deteriorated after her second daughter was born, but it still had a big impact on her.

Since starting dialysis, she feels her kidney disease has been disrupting almost every aspect of life at home with her family, and she worries she is not there enough for her daughters. Joanne feels tired and worries she isn't coping well. Sometimes she thinks her family would be better if she wasn't there.

Part 1. Struggling and getting stuck

Joanne has a lot of trouble following the diet restrictions that go with dialysis treatment. It's a big problem because she needs to lose some weight as she has gained a lot of weight in the last few years. She is so tired all the time that she just eats snack food to keep herself going. She finds the renal diet boring and doesn't want to make the changes the dieticians have suggested.

For Joanne, eating is a comfort and eases her anxiety about her family worries for a short while. It is also a nice way to relax with the family in the evenings so they often get takeaways. However, this is not helping her to lose weight. She also knows she is drinking too much fluid but when she feels thirsty she becomes angry about her situation and drinks more.

She is aware that she is feeling worse and not better since starting dialysis. Joanne knows she needs to lose weight and control her fluid intake to get the best results from dialysis. She also knows she has to avoid gaining weight between dialysis sessions and avoid taking too much salt with her food. Each time she comes to dialysis she finds she has gained more weight. In the short term, eating makes her feel better and helps her cope with a stressful situation, but long term it is compromising her health, increasing her fluid gains and she is gaining weight rather than losing it. She feels guilty about her weight and feels she is letting her family down.

Joanne's nurse suggests she needs some more specialist help.

Part 2. Managing difficult thoughts and feelings

Joanne was asked to meet with a specialist Dietician. She asked her to use a technique to sit with her feelings. She thought this would be a bit odd to do but agreed to try it out. She would just sit there and observe what she was feeling – a bit like she was a scientist and keep a diary. She did this over a few days and wrote down what she noticed. She was surprised to find that she felt lots of different feelings before she ate snack food or

suggested getting a takeaway for the family. She realised she often ate when she was tired, or upset, and often didn't feel hungry or thirsty at all. She would feel bad about letting her family down which is why she suggested a takeaway.

As Joanne became more aware of what her feelings were, she tried stopping before she ate or drank something, or suggested a takeaway, and to take five breaths and focus on what she was feeling.

Again, she was surprised to find this helped her stop and gave her space to think about whether she really wanted to eat or drink. When her difficult feelings came up, her usual impulse was to go to the fridge. Now, she waited and asked herself whether this was helping her go in the right direction.

As she sat with her feelings, she realised the bad feelings didn't get worse and actually reduced a bit after a while. She was able to think about making different choices about eating and drinking fluids.

Part 3. Who am I now? Living in the present

Joanne said being a good parent was important to her. She talked about how managing work, home, job and being a parent kept her busy all the time and so she rushed from one thing to another. The Dietician suggested Joanne could go to a class a Counsellor was running in the unit about Mindfulness. She explained this was about learning to slow down and not do things on automatic pilot. Joanne wasn't sure it would help but agreed to try it.

During the mindfulness session she was introduced to the idea of mindful eating. This involved eating something really slowly and noticing the experience with all her senses. She was surprised how much she experienced. She was used to eating quickly and not even noticing what she was eating. She decided to attend the next class and learn more about how mindfulness might help her generally. She was surprised that just slowing activities down helped her appreciate the experiences she was missing by being so busy. She started to become more aware of what she was feeling.

Part 4. Living with purpose and taking action

Joanne was asked by the Dietician to think about what was most important to her. She was clear that her family was really important to her and that she wanted her children to have fun times with her. 'Family' life and 'Fun' were top of her values list but she didn't feel like she was enjoying family life or having much fun. She recognised that her health had got worse since starting dialysis and that her health was also really important to her.

She realised that losing weight was going to involve making big changes at home. She decided to involve her family in cooking with her and asked the Dietician to help work on meal plans which followed the renal diet but were interesting and that she could adapt for the rest of the family. She asked them to work with her to agree a realistic goal for weight loss. In the past she had just tried to 'lose weight' and quickly gave up her 'diet' and started eating. She realised making small goals which she could achieve was a more helpful approach.

She had not ever exercised or played any sport, partly because she had often been ill. Her doctor was supportive of her increasing her activity gradually and she started walking more and going swimming with her children. At first it was really hard and she wanted to give up as she was so tired but she reminded herself why she was making this change and asked for support from her family when she was discouraged. Slowly as her fluid gains and weight improved she started to feel better and less tired. She was pleased to find she could have 'fun' with her 'family' and improve her health at the same time.

Story 3 – 'Margaret'

Margaret is 59. Her husband died from cancer a number of years ago. Their two children are grown up and are independent. Her son has left home but her daughter still lives at home but is hoping to move out in the near future. Previously, Margaret was a teacher and loved her job but had to retire early because of her illness.

Margaret has polycystic kidney disease, which is genetic, so she worries about her children. Her son was screened already, but her daughter says she does not want to know if she is affected and has chosen not to be tested.

Part 1. Struggling and getting stuck

Margaret delayed starting dialysis for as long as possible as she wanted to remain independent. She attends dialysis but hates dialysis needles being put in and gets very distressed each time she has this done. She initially found it painful and although she now has cream on before dialysis which helps her a bit with the pain, she has started to dread the thought of staff putting needles in her and gets very tearful. She is embarrassed that she gets so upset about having dialysis needles put in, and that she needs so much help and reassurance from the staff when that is happening.

Margaret really wants to be a good dialysis patient and give a positive image to her children about kidney disease. However, she often thinks back to how independent she was when she was working and that she now feels she is trapped. This makes her feel more and more fed up. When she is not at dialysis, she often stays in bed thinking about all her worries. She doesn't tell anyone how bad she is feeling and tells her children everything is going well.

In the short term, she thinks she is protecting her children from seeing how upset she is feeling. In the long term, she is feeling unhappy and isn't getting any help with the things that are worrying her or getting support to reduce her distress around needling.

Margaret's nurse asked her to sit down and talk about her struggles, as staff were becoming worried about her.

Part 2. Managing difficult thoughts and feelings

Margaret said she had all these worries in her head, she thought about them all the time, but she felt as if she could not talk about them. The nurse asked her to just stop and notice how many different thoughts she had in a minute. She was surprised how busy her mind was with worries about her situation and the future.

The nurse suggested it might help her if she got them out of her head. She suggested that every day she sit down and write for 15 minutes about everything that was worrying her and whatever she was feeling. The next step would be then to write down things that she could do to address some of the worries.

Margaret wasn't sure this would help but she decided to give it a go.

She found seeing everything laid out helped her to separate her worries. They were easier to look at and easier to think about when they were written down. She noticed that she was

telling herself what a burden she was because she couldn't cope on dialysis, and because she didn't want her family to find out she wasn't coping.

When worries cropped up, she would say to herself they could wait until the next time she sat down for her 'worry time', and this stopped her feeling quite so bad throughout the day.

She also started to think about what she would do with some of these worries rather than going to bed to hide from them. She wondered if she could get some help with having dialysis needles put in, so that she would find that less upsetting.

Part 3. Who am I now? Living in the present

The more Margaret wrote each day, the more she thought about how much she had seen herself as a strong, independent person. Even after her husband had passed away she had continued to work. This had always been important to her and this was why she had tried to put off starting dialysis. She had always seen herself as someone in charge at work and she found it really hard to stop working. She also found it difficult to be cared for by others as she had always liked to be in control. Her biggest fears had been that she would become a burden to her family and others. She realised that there had been changes in her life when she stopped working but also that she had got hooked into a story of being 'a burden' and had missed out all the things she still could do from her 'story'.

Her nurse asked her if she had told her family about her anxiety and upset about coming to dialysis. She admitted she hadn't told them because she didn't want to scare them in case they needed dialysis themselves. The nurse asked her how keeping her experiences from them was helping her or them in the future. She said if I was your family member, I would want to know if you were finding something difficult and try and help you with it and find a solution. Margaret hadn't looked at it this way.

Part 4. Living with purpose: taking action

Through her writing, Margaret had realised that being independent and in control were important values to her. One day she sat in the waiting room before dialysis talking to another patient. The person said they had started to get involved in her own care, and was hoping to train to do dialysis at home. Margaret was surprised as the person was older than her. They told her that she had recently started to put her own needles in.

Margaret decided to make a 'bold move' and asked the nurse if she could get involved in her care more. The nurse encouraged her to lay her hand on the nurse's hand as she needled her. When Margaret thought about taking a step forward and needling herself she noticed her mind was full of negative thoughts telling her 'you can't do this'. Her writing activity had showed her that her mind would worry about anything and warn her of potential problems to keep her safe. Rather than get hooked up in her negative thoughts she just noticed them but focused on what she could see, hear and feel around her. Her nurse said she didn't have to wait until she felt OK to try it but suggested she just give it a go. The next time she decided to do it even though she felt anxious and surprised herself that it was much easier to do than her mind was telling her.

She told her family about her anxiety and they started to pop in to see her on dialysis. They were supportive and wanted to find out all about the treatment. Margaret realised that only hearing positive things wasn't protecting them. What helped them was seeing that she had had some difficulties but they could be overcome. Margaret found getting involved in her own care and putting her own needles in helped her feel more independent and in control and actually hurt less as she got to know the best angle for her needles to go in.

As she started to feel better about dialysis, she started to do more generally. She decided to do some voluntary work in a school listening to children reading once a week and started to think about if dialysing at home was the right option for her.

Story 4 – 'David'

David is 73 and has always lived on his own. He retired from work as a car mechanic five years ago. He enjoyed his friendships with his work colleagues.

David had been planning to travel a lot during his retirement, but he had to cancel previously arranged travel plans at the time of going on to dialysis, and since starting dialysis has not managed to arrange any more holidays.

David has type 2 diabetes, which was what caused his kidney disease, and he feels guilty that he didn't look after his health better and potentially avoided some of his current health problems.

Part 1. Struggling and getting stuck

David has had a lot of problems with the transport to the dialysis unit. It often arrives late to pick him up, and then takes longer than he thinks it should to get to the hospital. By the time he arrives he is in a terrible mood, and sometimes lets off steam in the waiting room, shouting at the nurses or other patients about how badly organised the transport is.

David usually forgets to bring anything to do, so he is often bored and tense during dialysis. There is too much noise to watch TV, and he gets annoyed with the other patients and people around the unit, and he often just sits there thinking about how unfair everything is – he feels as if most people get picked up before him, and everyone seems to get treated better than he does. Sometimes he sleeps during dialysis but then he doesn't sleep at night.

In the short term, getting angry and letting off steam relieves some tension for David, but in the long term it doesn't help the dialysis go well, as the staff and other patients get tense and feel awkward being with David. David feels increasingly unhappy about his situation. The unit manager suggests to David that he could talk to a Psychologist about his angry outbursts.

Part 2. Managing difficult thoughts and feelings

The Psychologist suggested to David that all his thoughts were a bit like trains coming in and out of his head. He tried an exercise where he just noticed how many thoughts went through his head in a minute. Some just passed through but others he got caught up in. David realised 'everyone-gets-treated-better-than-me' had become a familiar 'thought train' that he often got on, especially during dialysis with nothing to do, and after a delayed journey to the hospital.

When the 'everyone-gets-treated-better-than-me' thought train came in, he got on it and stopped thinking about anything else. He remembered more and more examples of how other people get better treatment than he does. As he thinks around this he became more angry and resentful, and this made him snappy and irritable with other people. He can see people reacting negatively to him but can't stop himself thinking about all the ways his situation is unfair. He realised this really didn't help him at all.

The important thing he learnt was that he could notice this thought train. He could watch the train as it pulled up, but not get on it. Or if he got on it, he would say to himself 'there I go again on the 'everyone-gets-treated-better-than-me' train', and get off. It took lots of practice, but this meant he didn't have to spend all his time on dialysis thinking about how unfair his situation was, and he stopped getting so angry. When he wasn't so caught up in his thoughts he was able to stop and notice that lots of other people were waiting for transport as well as him. He also noticed that sometimes he was waiting to go on dialysis but so were other people. As he stopped being so cross he started to talk to other people. He found some people he got on with and asked the staff if he could sit near them on dialysis so he had someone to talk to.

Part 3. Who am I now? Living in the present

David had always kept busy. He had enjoyed practical hobbies like woodwork and was always helping friends mend their cars when he was not at work. He thought of himself as 'always on the go' and not someone to sit around. He wasn't much of a reader and hated sitting doing nothing. He felt dialysis was wasted time which he could be using to do things he enjoyed. He felt tied to dialysis and regretted he hadn't been able to follow his dreams of travelling. Deep down, he felt guilty about not taking as much care of his health and diabetes as he could have. He found it easier to be angry with everyone else than accept that he had choices about his situation going forward.

The Psychologist encouraged him to accept the things he couldn't change but to focus on what he could change. He had been so caught up in how much he had lost, he hadn't looked at what he could do to change his situation so this was a big challenge to him. However, David had always liked a challenge and had enjoyed solving problems at work so was willing to think about what steps he could take to change his situation.

Part 4. Living with purpose: taking action

David talked about how he valued keeping busy and trying new things, hence his plans had been to travel in retirement. The Psychologist asked him to look at what he could do on dialysis other than sleep (he had talked to the Psychologist about sleeping on dialysis during the day not helping him sleep at night and that in turn then didn't help his overall mood). David was asked to think about the things he had enjoyed doing which he had stopped doing and he could adapt to do on dialysis. He was also asked to think about new things he could do on dialysis. He recalled he had previously enjoyed watching comedy programmes and used to watch these in an evening if he wasn't seeing friends. He decided to get some DVD box sets of programmes he used to enjoy and brought them in to watch on a portable DVD player. He used to listen to the radio when he worked in the garage and realised he could listen to the radio and by using headphones he wasn't as bothered by the noise on the dialysis unit. Although he wasn't a reader, he joined the library and took out audiobooks which he started listening to during dialysis. He also started an audio course to learn Spanish. He found the time went quicker when he filled it with talking to other people, watching DVDs, listening to the radio, audiobooks and learning a language. He started to see dialysis as 'his time' where he could do activities he enjoyed rather than wasted time.

As he started to feel brighter in himself, David wondered about going on holiday. It had felt too difficult before but he talked to staff and realised that with some planning it was still possible to travel while undertaking dialysis treatment. He started to make plans for a holiday in Spain to try out his new language skills.

Thank you very much for reading these patient stories.

Please remember they are not real cases – they are fictitious cases based on lots of different people's experiences who have struggled to adjust to dialysis and are designed to illustrate some points about the kinds of difficulties some people experience and how they can overcome those difficulties and live better with dialysis.

Now you have read the stories, the researcher will get in contact with you to arrange a suitable time to speak with you either over the telephone or via Zoom video conferencing to capture your views and opinions about them. We also want to find out your thoughts about how we should present the stories in the next stage of the research e.g. films or written information. Thank you so much for taking the time to support our research.
