# Supplementary material for: Using patient feedback to adapt intervention materials based on acceptance and commitment therapy for people receiving renal dialysis
Source: BMC Urol. 2021 Nov 15;21:157. doi: 10.1186/s12894-021-00921-5 (PMC8591942; doi:10.1186/s12894-021-00921-5)
Supplement: Supplementary file 2 — Additional file 2. Dataset of participant feedback. [file 12894_2021_921_MOESM2_ESM.pdf]

Additional File 2. Dataset of participant feedback

Themes	Proposed modifications
1. The stories were credible.	“Generally I felt the stories, the characters were believable.” (Interview 3)<br>“Yes, they were very good actually and some of it is spot on.” (Interview 4)<br>“I found a little bit of my situation in each of them, and when I did that I thought, oh, so, how would I react and how would I behave and would that benefit me, you know.” (Interview 5)<br>“It's pretty much how things are I think.” (Interview 8)<br>“I was thinking this can't be made up.” (Interview 12)<br>“I know they’re fictional, but they probably could be real cases because people do suffer and like I say especially at the moment with things as they are um I think the mental health problems are far greater than perhaps they would've been.” (Interview 15)<br>“I could relate to all of them.” (Interview 18)<br>“To me they just read as though they were overly dramatic, it was overly stereotyped. They just didn't come across as real people to me.” (Interview 6)<br>“They were very, I think, probably stereotyped.” (Interview 17)<br>“I don't know why they have to be made up. I don't know why they can't just be real stories from real people.” (Interview 6)<br>“Yeah, but couldn't you get say a person like myself who wouldn’t mind, get their story which is actual facts.” (Interview 13)
2. Don’t sugar-coat dialysis.	“… there’s got to be one there whereby the outcome is the status quo I would’ve thought because not everyone’s gonna have a wonderful time doing dialysis and of all those stories that you’ve put forward, at the end of every story it was dialysis, great, I love it. And I don’t honestly think anyone on dialysis thinks that.” (Interview 1).<br>“Unfortunately, sugarcoating stories I don't think is gonna help some people because you know, some people need to be told you must do this otherwise you're gonna die. […] I'd want hard facts and the truth, whether it be good, whether it be bad, I just want to know where I stand.” (Interview 6)<br>“I found the first month or so absolutely horrific. I really did and I couldn't see anything in the stories that I could relate to, err regarding my initial start.” (Interview 17)

	“Where I would say I'm not sure the stories are realistic is that they presuppose a level of support, a dialogue, between hospital staff and the patient. Which I don't think is usually there at all.” (Interview 18)
3. Don’t always make the first thing they try work out.	“… the way that they work they sort of go down and then to a thing and then start climbing up the other side which is the way dialysis should be …” (Interview 2)<br>“Yeah, actually I like the four stages of each story, you know, their background, and then you know the approach and then the outcome so you know, sort of what's going wrong, how to deal with it, where to bring in help from and then you know moving forward.” (Interview 5)<br>“… at no point did the first suggested solution fail. […] it would be worth doing one whereby the first solution that maybe the renal psychologist or maybe the consultants suggested or maybe the nurse suggested didn’t work but because they’d made that effort they were then able to go and talk to someone to find another way to try and sort it out” (Interview 1)<br>“… a psychologist would probably try various different methods with their patients and obviously different techniques work for different people erm so yeah so you could have them, you know, saying that they tried this or they tried that but then they found that this was the best method that helped them the most.” (Interview 3)
4. Psychologists are not so available.	“… in my dialysis unit we have a renal counsellor but we don’t really have a psychologist. […] when I went to a psychologist I had to do that all privately.” (Interview 3)<br>“I was like really, really low, um and the guy in there said to me, like oh I'll refer you to a psychologist, but I never got referred to one.” (Interview 7)<br>“I've never seen a psychiatrist or psychologist. Nobody's ever said you could talk to a psychiatrist. Nobody's ever said you could talk to a counselor.” (Interview 18)<br>“I think it's a little bit idealistic at the moment to you know to put forward these stories, which are basically solved by a patient talking to um, somebody who's got the time and the ability to guide them um into these therapists and I mean the nurses don't have the time, do they.” (Interview 18)

5. Involve other people.	"… what is it like to live with a dialysis patient because it's not a journey just for one person, it could be a journey for the partner, or family member that helps, or friend that helps." (Interview 4)<br>"I think help from other people is very, very important, whether you've got a, uh, a partner or family at home, or you can see a professional. […[emphasizing the aspects of help and not going and doing it yourself are very important, very important, actually." (Interview 5)
6. Realism in individual stories.	**"**I'm a Hindu and you know when I had kidney failure for my parents it was hard to take and being an Indian is very different to being a Caucasian in all aspects of life and I think that's with whether you're a Hindu, Muslim, a Sikh or whatever I think on general broad terms in an Asian community we have a very different way of life …" (Interview 4)<br>"… we need to show that you can have local holidays in your country, you can go shopping, you can go for dinner, these things are all positive and we need to show that …" (Interview 4)<br>"… you have to look at other things to look forward to. Like going out for a meal or something you know or going out for a day when you're not on dialysis." (Interview 13)<br>"Kieran's 25 and he lives at home so although he's concerned about work I didn't feel he needed to worry about it as much cus he was living at home with his parents. If he was a single person living, you know, and had his own house maybe a bit slightly older and had his own house then I could understand the concern for his work more because he's got to pay for a mortgage or rent and this sort of thing and buy his own food and this sort of thing." (Interview 3)<br>"You know, his work calling him in and he just sort of jumped off dialysis and went to work. I thought it was a bit strange because my impression was that anybody starting dialysis would necessarily tell their employer, and it's a very rare employer that would sort of expect someone to leave dialysis and go to work." (Interview 5)<br>"I think workplaces would understand that, and I don't think they would be so pushy to um, you know, call him while, call someone while they're on a session and get them to work, and especially like quite frequently." (Interview 7)<br>"… I was just wondering why he wasn't considered for evening dialysis so because […] I work so I work 9 to 5 and then go to dialysis after work […] the

	nurses might suggest for him to do evening dialysis so then he could actually work."(Interview 3)<br>"I know like a lot of people that are at work, um like that go to the dialysis unit, you know, I'm on the late shift now, so most of the people in there work and they're all quite supportive of them and their disease." (Interview 7)<br>"I didn't think his employers were very understanding. But that's probably through ignorance because unless you actually know somebody who's got the problem … […] it was probably through ignorance that his bosses didn't understand what was happening to him, didn't realise that it was vital that he had treatment or else he'd die. And also probably some colleagues as well are not very sympathetic because they don't understand why you keep having to have time off and go for treatment and what it means and to somebody who's not really understanding it they probably think you're just skiving somewhere just to get some time off." (Interview 15)<br>"I think it's a big help actually because if you you know how other people have reacted to different situations and how they have got themselves through it, if they've got themselves through it. It's gonna be, it's a big help, I think the guidance and put in you know that, you can get through these things and you can work over things and yeah so it's got to be a positive." (Interview 8)<br>"…I felt it was quite sad but I I've been, I've been there, done that, um that's as much as I can say." (Interview 12)<br>"… how many people would risk giving up a full-time job when they're fairly young to just study while on dialysis, I don't know. I think it was fine, but I found it a tiny bit unbelievable that he would just give up work like that." (Interview 5)<br>"At my dialysis center I don't see anybody doing anything except sleep or watch the TV generally, and I think that for Kieran, this guy to actually study during dialysis I found that stretched the imagination a little bit …" (interview 5)<br>"[The story] where they're struggling with the fact that they have to do this and it's not very pleasant, it's the woman who's got the two kids … […] that story rings true, it rings very very true with a lot of people … So there's a big problem with people not understanding what the treatment is, so even though you include people in the treatment they just don't get it." (interview 1)

	"… those characters I identified with a couple of them, you know, you can you feel a bit sorry for yourself, or down and so you seek comfort in junk food or something. You know it's not good for you but you just you can't be bothered. You don't want to be good to yourself. You don't want to eat healthy salads because you're feeling sorry for yourself so you see comfort in stuff that you shouldn't do." (Interview 5)<br>"I think one of the best outcomes you can have on dialysis is if you do everything yourself … … so that might be worth putting in the one you talk about the girl who's needling herself. And even if you're not going to needle yourself because you can't needle yourself because of fear or whatever else, just coming in and lining your machine up and getting everything ready is an element of control that makes life a lot easier, so that might be worth adding into that story with her." (Interview 1)<br>"The idea that people would just pop into dialysis, because it's definitely discouraged at my dialysis center, you know, unless someone's a family member [and] is coming in to actually help to dialyse you. But the idea that the dialysis center would be like a hospital where you have visiting hours, I don't know about other dialysis centers, but I found that a very strange thing in the story that family started to pop in. […[I'd certainly tweak that sentence. You know, family popped in. I would change it to something like, you know, family started to ask more questions and I don't know." (Interview 5)<br>"I would say by all means really get involved in your dialysis, needle yourself, you know, cannulate yourself and there you get back that independence. But as to doing it at home, um not necessarily encouraged because you lose a lot of the the social interaction that you get from dialysis, and sometimes when you're feeling down I could imagine you know if you're sort of having to get all of these materials, get the bag, stick the needles in yourself, and sit there for four hours it's, you know, sometimes you need to be having somebody help you and interacting with them and stuff like that. I wouldn't go for home dialysis now, and that's a real surprise to myself." (Interview 5)<br>"…she's trying to set a lot of positive image about it, but I think with that like the reality of it is like there's not a lot of positive things about having kidney disease like. People have got to know the truth about it because you know, if anyone ever gets it, you know like her children, for instance they'll probably

	think, oh, like it's fine my mum set like a really positive thing about it but to be honest, it's quite a hard disease to have. […] she doesn't really need to set a positive disease for it because if her children do get it, then you know they'll find out for themselves that it's quite a hard, quite hard thing to deal with." (Interview 7)<br>"Initially I was like one of your characters like one of the elderly characters in those four, I was very used to being independent so I hated going to dialysis …" (Interview 5)<br>"Yeah, well I think that and because in the early days I was very similar, like I was very impatient and very intolerant at the start. Err, I'm not now I've mellowed considerably." (Interview 17)<br>"… he was a car mechanic, retired, but he didn't seem to have a car in itself, so we went with transport. […] in his case I just thought it was a bit strange that he was, it was, uh, it was an inappropriate choice of profession. He's a car mechanic and likes to fix peoples cars, but he doesn't drive himself." (Interview 5)<br>"David was taking DVD box sets in and a portable DVD player, I thought that might be a bit outdated now but that's all. I think units tend to have Wi-Fi connections, I mean I'm not too I know ours does but ours is quite a brand new unit so some might not have that but if they do have Wi-Fi then yano you've got access to things like Netflix, iPlayer, these sorts of things, audio books on your tablets these days so yeah its quite good." (Interview 3)
7. Focus more on the techniques.	"I liked the one about the trains and getting on the old um, you know, I'm cross with everyone train and don't keep doing that sort of thing." (Interview 3)<br>"And also the writing down technique as well I've heard that before, being able to write your feelings down so you understand how you're reacting at a time and then yano you might look back on it the next day and realise oh that was silly why were you sort of thinking that way." (Interview 3)<br>"I mean they were there was a different strategy for each person and yet the strategies were all they all went back to self-acceptance or self-awareness and an understanding of what was very challenging for them. […] so it was quite clear that the strategies used were, well-tailored towards the individual because obviously you wouldn't use the same strategy for all of them, and they

	were very different personalities, but all facing quite a quite a big challenge.” (Interview 5)<br>“I think that was in one of their stories, you know, like gathering your thoughts and like tackling them one at a time and not altogether and just trying to think as positive as you can, because like you've got, you've gotta look ahead like this is not gonna be forever, this is just like for now. Um, I think another one was set like setting goals for themselves.” (Interview 7)<br>“there was actually one in there that I picked up on and I think it was the thinking positive one. Um, because like this isn't forever, but like you know, you can get yourself in quite of a rut if you don't, you know, think positive, yeah, that was quite a good one.” (Interview 7)<br>“Writing down my thoughts, I won’t know, it did help me, I won’t know if it will help anybody else. I won’t know that.” (Interview 11)<br>“I appreciated that because I keep a diary, that's one of the things I learnt at previous dialysis was to keep a diary.” (Interview 12)<br>“… a lot of the people you’re dealing with in hospital, I think they would have to be shown these techniques very, very sort of gently and carefully… And that would all take a lot of time and that's the basic problem, you know.” (Interview 18).
8. Use video format.	“As text it all, it was just too long. But if you are planning on doing it as a video then it wouldn’t really matter …” (Interview 6)<br>“Reading like a lot of words like would not be helpful for me because I wouldn't like be able to think about it properly, like I'd read it, but it just would not go in.” (Interview 7)<br>“I think four was enough.” (Interview 10)<br>“I think it could be quite interesting to have sort of little video clips of people talking about their stories. […] “little video clips or something that you could watch if you were struggling or yano and listen to other peoples stories and it might help to you know give you ideas about how to um improve your problems.” (Interview 3)<br>“Some people like to just stare at the screen so I think I'd like to see like four characters coming on and watching them you know, on a sort of video type thing, you know if the characters were believable and everything, I'd like to watch that” (Interview 5)

	"I struggle to get through it to be honest with you, you know, so I'm thinking it's much easier to just watch, watch something as a video." (Interview 6)<br>"Reading like a lot of words like would not be helpful for me" (Interview 8)<br>"I do think actually as well that sort of visual video or audio or whatever is a lot better than written" (interview 8)<br>"Yes anything like that rather than having to read something because reading it, it doesn't seem real, whereas somebody talking to you it seems more real, I think." (Interview 12)<br>"Erm I think you'd be better off if you could get the people who are actually on dialysis to do it rather than an actor …" (Interview 1)<br>"I think to be honest the best, the perfect scenario would be for a patient to talk about their own situation, but now that you have scripts and stories which are a range of different people's experience I guess it's better to have a, uh, a good actor." (Interview 5)<br>"I mean you could try something humorous with animation …" (Interview 1)<br>"Provide a tablet and this clamp and you can just, any patient can then watch it, clamp it to the table …" (Interview 2).<br>"I'd watch it during dialysis definitely." (Interview 7)<br>"… then if they could watch something at home as well, because then if they've got, if they're living with family and stuff, wives, kids or whatever, or parents or whatever, then they can share ideas can't they." (Interview 8)
